# Genetics of Callous-Unemotional Behavior in Children

**DOI:** 10.1371/journal.pone.0065789

**Published:** 2013-07-09

**Authors:** Essi Viding, Thomas S. Price, Sara R. Jaffee, Maciej Trzaskowski, Oliver S. P. Davis, Emma L. Meaburn, Claire M. A. Haworth, Robert Plomin

**Affiliations:** 1 King’s College London, Medical Research Council Social, Genetic and Developmental Psychiatry Centre, Institute of Psychiatry, De Crespigny Park, London, United Kingdom; 2 Division of Psychology and Language Sciences, University College London, Gower St, London, United Kingdom; 3 Institute for Translational Medicine and Therapeutics, University of Pennsylvania School of Medicine, Philadelphia, Pennsylvania, United States of America; 4 Department of Psychology, University of Pennsylvania, Philadelphia, Pennsylvania, United States of America; 5 Department of Psychological Sciences, Birkbeck, University of London, Malet Street, London, United Kingdom; Yale University, United States of America

## Abstract

Callous-unemotional behavior (CU) is currently under consideration as a subtyping index for conduct disorder diagnosis. Twin studies routinely estimate the heritability of CU as greater than 50%. It is now possible to estimate genetic influence using DNA alone from samples of unrelated individuals, not relying on the assumptions of the twin method. Here we use this new DNA method (implemented in a software package called *Genome-wide Complex Trait Analysis*, *GCTA*) for the first time to estimate genetic influence on CU. We also report the first genome-wide association (GWA) study of CU as a quantitative trait. We compare these DNA results to those from twin analyses using the same measure and the same community sample of 2,930 children rated by their teachers at ages 7, 9 and 12. GCTA estimates of heritability were near zero, even though twin analysis of CU in this sample confirmed the high heritability of CU reported in the literature, and even though GCTA estimates of heritability were substantial for cognitive and anthropological traits in this sample. No significant associations were found in GWA analysis, which, like GCTA, only detects additive effects of common DNA variants. The phrase ‘missing heritability’ was coined to refer to the gap between variance associated with DNA variants identified in GWA studies versus twin study heritability. However, GCTA heritability, not twin study heritability, is the ceiling for GWA studies because both GCTA and GWA are limited to the overall additive effects of common DNA variants, whereas twin studies are not. This GCTA ceiling is very low for CU in our study, despite its high twin study heritability estimate. The gap between GCTA and twin study heritabilities will make it challenging to identify genes responsible for the heritability of CU.

## Introduction

Callous-unemotional behavior (CU) – defined by low levels of empathy, absence of guilt and emotional unresponsiveness – is currently under consideration as a subtyping index for conduct disorder in DSM-V [1] and may have independent diagnostic value, even in the absence of a conduct disorder diagnosis [2] [3] [4] [5]. CU often occurs in the presence of conduct problems (see e.g. [6] [7]) and predicts vulnerability to psychopathy in adulthood [8]. Several longitudinal studies of large community samples now suggest that CU can also occur in the absence of clinical levels of conduct problems (e.g. [2] [3] [5] [7]). In the cases where current levels of conduct problems do not reach clinical levels in children with CU, sub-clinical levels of or later developing conduct problems are typically observed [2] [3] [5]. In addition, and perhaps more interestingly, individuals with CU and non-clinical levels of conduct problems commonly show elevated levels of other types of impairment, including poor peer relationships, low pro-sociality, and increased hyperactivity [2] [3] [5] [7]. CU therefore has the potential to serve as a useful clinical indicator for psychiatric vulnerability and psychosocial maladjustment, in addition to its utility in subtyping children with conduct disorder.

Individual differences in CU are estimated to be moderately to strongly heritable using the twin design that compares resemblance in monozygotic (MZ) twins and dizygotic (DZ) twins in community samples of children and adolescents (heritability estimates from .45–.67; see [9] for a recent review). Having elevated levels of CU is strongly heritable in childhood regardless of whether CU traits are accompanied by conduct problems or not [10]. Twin studies suggest that there is considerable overlap in the genes that influence CU and conduct/externalizing problems, but that there are also unique genetic influences on CU [11] [12] [13]; consistent with the finding that high levels of CU have been observed in the absence of clinical levels of conduct problems [3]. CU is moderately to strongly stable during childhood [14] and twin studies suggest that stability in CU/psychopathic behavior is driven by genetic influences [15] [16]. This finding of genetic stability led us to conduct a new twin analysis focused on a composite measure of CU across ages 7, 9 and 12 in an attempt to create a genetically enriched measure of CU.

The high heritability of CU has led to the first attempts to identify some of the genes responsible for its heritability. Only a handful of published candidate gene association studies to date have focused on CU in *children or adolescents* [17] [18] [19] [20] [21] [22] [23] [24] [25]. However, the sample sizes have been smaller than 200, fewer than half a dozen candidate genes have been investigated, and the results of these studies have been mixed and contradictory. Even adequately powered candidate gene studies have a poor record for replication [26] [27]. The poor track record for candidate gene studies has been one reason why the field has moved towards systematic genome-wide association (GWA) studies [28]. GWA studies were made possible by the development of commercially available DNA arrays that can genotype hundreds of thousands of single-nucleotide polymorphisms (SNPs) inexpensively [29]. The SNPs on DNA arrays are distributed across the 23 pairs of chromosomes in order to tag all common DNA sequence variation in the genome. Each SNP is correlated with the target trait as in a candidate gene study but GWA systematically scans the entire genome for associations and, crucially, corrects significance levels for multiple testing so that the accepted level of significance for GWA studies is p<.00000005.

Genome-wide association (GWA) studies for psychiatric phenotypes have shown that genome-wide “hits” are often in genes that were not previously hypothesized to influence the phenotype or not in traditional genes at all [28] [29] [30]. To our knowledge, only one GWA study incorporating CU has been published to date [31], which was from our group and focused on children with a combination of high CU and conduct problems, using allele frequencies estimated from DNA pooled across children in the high group and a control group rather than genotyping each child individually. Although our DNA pooling GWA study of the extremes had power to detect genes of large effect size, none of our associations reached genome-wide significance. The present report is the first standard genomewide association (GWA) study of individual differences in CU assessed as a quantitative trait – our previous GWA study investigated the extremes of co-occurring CU and conduct problems using DNA pooling.

After more than a thousand published GWA studies across the life sciences [32], we now know that the largest effect sizes for GWA associations are likely to be very small, accounting for less than 1% of the variance of quantitative traits [33]. Although it is nonetheless useful to exclude the possibility of large effect sizes – which were found for example in the first GWA studies on macular degeneration [34] – it now seems unlikely that our GWA sample of about 3000 children would have the power to identify genome-wide significant associations of the expected small effect size. Much has been written about ‘missing heritability’ [35], the gap between GWA-identified associations and heritability as estimated in twin studies, with rare variants and non-additive effects as the most likely culprits [36]. GWA studies have been limited to the common SNPs used on commercially available DNA arrays and to additive effects of SNPs considered individually rather than multiply as they interact in their effect on the phenotype.

Another reason for the missing heritability gap could be that twin studies have overestimated heritability. A new method, implemented in a software package called Genome-wide Complex Trait Analysis (GCTA), uses DNA alone to estimate genetic influence from samples of unrelated individuals, not relying on the assumptions of the twin method [37] [38]. GCTA does not identify specific genes associated with traits. Instead, it uses chance similarity across hundreds of thousands of SNPs to predict phenotypic similarity pair by pair in a large sample of unrelated individuals. The essence of GCTA is to estimate genetic influence on a trait by predicting phenotypic similarity for each pair of individuals in the sample from their total SNP similarity. In contrast to the twin method, which estimates heritability by comparing phenotypic similarity of identical and fraternal twin pairs, whose genetic similarity is roughly 1.00 and .50, respectively, GCTA relies on comparisons of pairs of individuals whose genetic similarity varies from .00 to .02. GCTA extracts this tiny genetic signal from the noise of hundreds of thousands of SNPs using the massive information available from a matrix of thousands of individuals, each compared pair by pair with every other individual in the sample; for example, the 3,000-plus individuals in the present sample provided nearly 5 million pairwise comparisons.

GCTA genetic similarity is not limited to the genotyped SNPs themselves, but also includes unknown causal variants to the extent that they are correlated with the SNPs. Mendel’s second law of inheritance is that genes (as they are now called) are inherited independently (a phenomenon now called linkage equilibrium), but Mendel did not know that genes can be on the same chromosome, in which case they are not inherited independently (linkage disequilibrium). This violation of Mendel’s second law is complicated by the fact that during meiosis, on average each pair of chromosomes – one from the mother and one from the father – crosses over (recombines) once; in the population, genes on the same chromosome are separated by this process of recombination to the extent that they are not close together on the chromosome. GCTA provides a lower-limit estimate of heritability because it misses genetic influence due to causal variants that are not highly correlated with the common SNPs on genotyping arrays.

A difference between GCTA estimates and twin-study estimates of heritability is that GCTA only estimates additive genetic effects, whereas the twin method captures nonadditive as well as additive genetic effects. Additive genetic effects are caused by the independent effects of alleles, which add up in their effect on a trait; nonadditive genetic effects are those that interact. Because GCTA adds up the effect of each SNP, it does not include gene-gene interaction effects; the twin method captures nonadditive as well as additive genetic effects because the DNA sequence of identical twins is virtually identical and thus they share all genetic effects, including nonadditive ones (see [39] for details). GCTA has been used to estimate heritability as captured by genotyping arrays for height [37], weight [40], psychiatric and other medical disorders [41] [42] [43], and personality [44]. We have used GCTA to estimate heritability for cognitive abilities using DNA alone and to compare these results to twin study heritability estimates from the same sample using the same measures at the same ages [45].

However, GCTA offers far more than a check on twin study heritability estimates – it provides important clues about missing heritability. Because GCTA estimates of genetic influence are limited in the same way as GWA studies to the additive effects of common DNA variants, GCTA will underestimate twin study heritability to the extent that nonadditive effects or rare variants are influential. Moreover, for this same reason, GCTA estimates of heritability rather than twin study estimates of heritability create a ceiling for GWA attempts to identify associations. Here we report the first GCTA estimate of genetic influence and compare it to a twin study heritability estimate using the same measure in the same sample in order to increase the precision of the comparison between them.

In summary, the overall aim of this research was to compare twin study heritability to GCTA heritability and to the results of GWA for CU assessed as a quantitative trait. The comparison of these three components of genetic influence has important implications for finding missing heritability.

## Materials and Methods

### Ethics Statement

Ethical authorization, including authorization to work with children, was given by The Joint South London and Maudsley and the Institute of Psychiatry Research Ethics Committee (05/Q0706/228). Parents were given a letter describing the general purpose of the study and written parental consent was required. It was made clear that participation was voluntary and participants could withdraw from the study whenever they wished.

### Sample

The sample was drawn from the Twins Early Development Study (TEDS), a multivariate longitudinal study which recruited over 11,000 twin pairs born in England and Wales in 1994, 1995 and 1996 [46], whose families are representative of the UK population [47]. Twins with severe medical problems or severe birth complications or whose zygosity could not be determined were excluded from the sample. To decrease heterogeneity of ancestry, the sample was restricted to families who identified themselves as white and whose first language was English.

In order to make our twin sample as comparable as possible to our GCTA and GWA samples, we selected those twin pairs for whom one member of the twin pair was chosen for the GCTA and GWA analyses. For GCTA and the discovery sample of the GWA analysis, we included unrelated individuals by selecting only one member of each twin pair for whom GWA genotyping and CU data were available. For the GCTA analysis, we verified that the unrelated individuals were less genetically related than fourth-degree relatives (genetic relatedness >.025), the standard GCTA exclusion criterion.

Based on these selection criteria, our twin analyses included 1099 MZ pairs and 1787 DZ pairs. Our GCTA and GWA discovery sample included 2,930 children; the slightly smaller number of twin pairs was caused by twin pairs for whom the co-twin did not have CU data.

### Genotyping Protocol

DNA was extracted from buccal cheek swabs and sent to the Wellcome Trust Sanger Institute, Hinxton, UK for genotyping as part of the Wellcome Trust Case Control Consortium 2 (https://www.wtccc.org.uk/ccc2/). A total of 3,747 DNA samples from unrelated children in TEDS were sent for genome-wide DNA array genotyping used in our GCTA and GWA analyses. In total, 3,665 samples were successfully hybridized to Affymetrix GeneChip 6.0 SNP genotyping arrays (http://www.affymetrix.com/support/technical/datasheets/genomewide_snp6_datasheet.pdf) using experimental protocols recommended by the manufacturer (Affymetrix Inc., Santa Clara, CA). The raw image data from the arrays were normalized and pre-processed according to the manufacturer’s guidelines (http://www.affymetrix.com/support/downloads/manuals/genomewidesnp6_manual.pdf).

Genotypes for the Affymetrix arrays were called using CHIAMO (https://mathgen.stats.ox.ac.uk/genetics_software/chiamo/chiamo.html). Where there was a sufficient quantity of DNA, samples were also re-genotyped on a panel of 30 SNPs (including 26 autosomal SNPs present on the Affymetrix array, and 4 SNPs on the X chromosome to verify gender) using the Sequenom iPlex Gold assay (Sequenom Inc., San Diego, CA).

### Quality Control: Samples

We identified and removed samples whose genome-wide patterns of diversity differed from those of the collection at large, interpreting these differences as possibly due to biases or artifacts. Outlying individuals were identified on the basis of call rate, heterozygosity, relatedness and ancestry using a Bayesian clustering approach [48].

To obtain a set of putatively unrelated individuals we used a hidden Markov model (HMM) to infer identify by descent along the genome between pairs of individuals. Among pairs of closely related individuals, we excluded the member of the pair with the lowest call rate, iteratively repeating this procedure to obtain a set of individuals with pairwise identity by descent less than 5% [48]. Of the individuals genotyped, samples were excluded because of low call rate or heterozygosity outliers (377), unusual hybridization intensity (9), atypical population ancestry (59), sample duplication or relatedness to other sample members (83), and gender mismatches (13). In addition, 54 samples were excluded because fewer than 90% of genotypes were called identically on the genomewide array and Sequenom panel. The remaining samples were consistent with previous genotyping. In total, 513 samples were excluded by these quality control criteria. The remaining sample of 3,152 individuals included 1,446 males and 1,706 females. Of this sample, 2,930 children had valid data for CU at age 7, 9, or 12, and 2,140 had data at two or more ages.

### Quality Control: SNPs

An index of information (Fisher) for the allele frequency at each of 932,533 called SNPs was calculated using SNPTEST version 2.1.1 [49]. Autosomal SNPs were excluded if this information index was below 0.975, if the minor allele frequency was less than 1%, if greater than 2% of genotype data were missing, or if the Hardy Weinberg *p*-value was lower than 10^−20^. Association between the SNP and the plate on which samples were genotyped was calculated and SNPs with a plate effect *p*-value less than 10^−6^ were also excluded. In addition, SNPs were manually filtered for call quality by visual inspection of the hybridization intensity plots using EVOKER software (http://sourceforge.net/projects/evoker/). The above filters removed 22.7% of the SNPs, leaving 699,388 autosomal SNPs for further analysis.

### SNP Imputation

In order to increase the number of SNPs used in our GCTA and GWA analyses, imputation was carried out using the IMPUTE version 2 software [50] on the genotype data after application of quality control procedures, using a two-stage approach with both a haploid reference panel and a diploid reference panel. For the haploid reference panel we used HapMap phase II and III SNP data on the 120 unrelated CEU trios. 5,175 WTCCC2 controls were genotyped on both Affymetrix 6.0 and Illumina Human1.2M-Duo arrays (Illumina Inc., La Jolla, CA), and these were used for the diploid reference panel. Imputed SNPs were retained for analysis if they were genotyped using the Affymetrix 6.0 array, if they were genotyped using the Illumina Human1.2M-Duo array and obtained an information score ≥0.90, or if they were imputed and obtained an information score ≥0.98. Using these criteria, 1,024,929 imputed SNPs were retained for the GCTA and GWA analyses, in addition to the 699,388 measured SNPs described above.

### CU Trait Measures

CU at 7, 9, and 12 years of age was assessed by each child’s school teacher using a paper (or at 12, online) questionnaire. Teacher ratings were obtained towards the end of the academic year when the class teacher had known the child for most of the academic year. In the U.K. there are no systematic differences with regard to placing mono- versus dizygotic twins to same or different classes. The percentage of twins rated by the same teacher is 65% at age 7, 58% at age 9, and 33% at age 12.

Teachers are familiar with a broad range of children and have expertise regarding normative child development. Teacher ratings have been found to show higher internal consistency and stability than parent ratings [51], and twin analyses indicate that teacher ratings are free of rater bias typically found in parent ratings [52]. In line with this, teacher ratings for CU show better internal consistency (e.g. α = .74 at age 7), indicating reliable detection of the latent construct of interest; parent ratings of CU show much poorer levels of internal consistency (e.g. α = .45 at age 7). Finally, the means and variances for the CU scale are typically lower for parents than for teachers, indicating that parents are poorer at discriminating children high in CU [53]. These problems with the parent rating scales led us to focus on the teacher ratings. The CU score was calculated as the total for seven items used in previous heritability analyses of CU (e.g. [10] [13]). These were original Antisocial Process Screening Device [54] CU items (e.g. ‘Does not show feelings or emotions’) or Strengths and Difficulties Questionnaire [55] items selected to reflect CU (e.g. ‘Considerate of other people’s feelings’ (reverse scored)). The sampling frame for CU at 7 and 12 included all children in TEDS. The sampling frame for CU at 9 included only children born between January 1994 and August 1995. For the purposes of twin and GCTA analysis, we calculated a composite variable from the mean of available teacher reports of CU at 7, 9, and 12 years. This composite required that at least one measurement be non-missing.

### Statistical Analysis: Twin

MZ and DZ twin intraclass correlations were calculated and standard twin model-fitting was used to estimate additive genetic (A), common or shared environment (C), and residual or non-shared environment (E) [39]. Although twin model-fitting is usually referred to as ACE models, in fact the twin design – unlike GCTA, discussed in the next section – can include non-additive as well as additive genetic effects. In quantitative genetics, estimates of heritability that include non-additive as well as additive genetic effects is called *broad heritability*, in contrast to *narrow heritability*, which is limited to additive genetic effects. In twin analysis, the additive genetic model assumes that DZ twins are half as similar as MZ twins because the genetic relatedness of DZ twins is 50% for additive genetic effects, whereas the relatedness of MZ twins is 100%. This twofold greater genetic resemblance of MZ as compared to DZ twins is the reason why heritability is often estimated by doubling the difference between MZ and DZ correlations. (For example, MZ and DZ correlations of 0.80 and 0.40, respectively, imply 80% heritability).

In contrast, the hallmark of non-additive genetic effects is that the DZ correlation is less than half the MZ correlation because epistatic (inter-locus) gene-gene interactions scarcely contribute to DZ similarity but are shared entirely by MZ twins. If non-additive genetic effects are important, the twin method will detect these effects, although its ability to estimate these effects is limited. For example, if MZ and DZ twin correlations are 0.80 vs. 0.20, respectively, simply doubling the difference between MZ and DZ correlations – an additive genetic model which would be inappropriate given the non-additive pattern of twin correlations – would yield a heritability estimate of 120%. However, heritability cannot exceed the MZ twin correlation, so that the heritability estimate in this example would be constrained to be 80%. Model-fitting would show that the ACE model does not fit the data in this example. An allowance is made for non-additivity in a twin model called *ADE*, in which ‘*D*’ refers to dominance (intra-locus allele-allele interaction). In the ADE model, dominance discounts DZ resemblance from 50% for the A parameter to 25% for the D parameter. However, this adjustment does not cover the extreme epistatic case in which the DZ correlation could be zero despite a high MZ correlation. However, even in this extreme case – for example an MZ correlation of 0.80 and a DZ correlation of 0.00 – twin model-fitting would detect genetic influence and would cap the heritability estimate at 80%, as suggested by the MZ correlation of 0.80.

In summary, the twin design can detect the presence of non-additive genetic effects, although it is limited in its ability to distinguish additive and non-additive genetic effects. Greater detail about distinguishing additive and non-additive genetic variance in twin designs is available (e.g. [39]). As is usual in twin analyses, residualized scores were used that were independent of age and sex because age and sex are perfectly correlated across pairs, which would be misinterpreted as C in twin analyses. The OpenMx package for R was used for twin maximum-likelihood model-fitting using full-information matrices [56].

### Statistical analysis: GCTA

We used the software package Genome-wide Complex Trait Analysis (GCTA; [38]) to estimate genetic influence from pair-by-pair similarity across all of the SNPs on the DNA array. We applied GCTA analysis to a composite variable from the mean of available teacher reports of CU at 7, 9, and 12 years, the same variable used in the twin analysis. This univariate phenotype was submitted to GCTA [38] in order to estimate by restricted maximum likelihood the proportion of variance explained by the genome-wide panel of SNPs. Both genotyped and imputed SNPs were included in the analysis. Individuals were deleted from the analysis if their estimated relatedness with another member of the dataset exceeded 0.025. Sex, birth year/school year cohort, and 8 principal components of the genotype data were included as covariates in the GCTA analysis.

### Statistical Analysis: GWA

Genome-wide association (GWA) analysis was conducted using a linear regression approach implemented in SNPTEST v2.0 [57] under an additive model. This approach uses a frequentist method to account for uncertainty of genotype information [49]. Because even small differences in allelic frequency within subgroups in the population can generate false-positive results, eight principal components representing population ancestry were used to control for population stratification. Sex and DNA sample plate number were also included as covariates. Results were visualized using Manhattan plots, quantile-quantile (Q-Q) plots, and genotype-phenotype plots, generated in R [58]; a regional association plot created using LocusZoom [59].

Following SNP quality control and SNP imputations, described earlier, we performed several preliminary analyses prior to GWA analysis. First, principal component analysis (PCA) was used to attenuate GWA biases due to population structure. PCA was conducted on a subset of 105,556 autosomal SNPs post QC, selected after pruning to remove SNPs in high linkage disequilibrium (*r* ^2^>0.2) and to exclude high linkage disequilibrium genomic regions so as to ensure that only genome-wide effects were detected [60]. Application of the Tracy-Widom test indicated that eight principal components were significant using a threshold of *p*<0.05. We caution that the inclusion of principal components as covariates may not be sufficient to remove biases in estimation due to population structure [61]. Our second preliminary analysis involved normalizing CU trait scores by transforming the ranked data to the quantiles of a standard Normal distribution using the van der Waerden transformation [62], and taking the residuals after regressing the resulting score on age at measurement.

For the GWA analysis, each autosomal SNP was tested for association with CU at 7, 9, and 12 using a multivariate method that was similar in its essentials to the test of a longitudinal composite but required slightly less restrictive statistical assumptions. Using a linear regression framework, we calculated score statistics to test a hypothesis that the SNP had an equal effect on CU at each age. The test evaluated a single parameter and hence had 1 degree of freedom. Sex, birth year/school year cohort, and the first eight principal components of the genotype data were included as covariates in the regression model. The statistical framework was able to account for missingness in the outcome variables as a function of these covariates, assuming that data for these covariates were missing at random [63]. The test was implemented as a custom library for R (http://www.cran.r-project.org).

Probability values were adjusted by genomic control [64] separately for genotyped SNPs, SNPs that were genotyped in the WTCCC2 controls and imputed in the TEDS sample, and SNPs that were imputed in both WTCCC2 controls and the TEDS sample.

## Results

### Twin analysis

Tables 1–3 present twin correlations and the results of model-fitting analyses for the composite CU trait. The difference in the MZ and DZ twin correlations suggests substantial genetic influence. The DZ correlation is about half the MZ correlation, suggesting no influence of shared environment or non-additive genetic variance, unless these two factors mask each other’s effect. Model-fitting results confirm the results gleaned from the MZ and DZ twin correlations. A model that includes only A and E, excluding C, fits the data best. Model-fitting parameter estimates for the full ACE model indicate substantial heritability (0.64 ± 0.03) and negligible shared environmental influence (0.00 ± 0.02).

**Table 1:** Twin correlations and model-fitting results for a callous unemotional (CU) trait longitudinal composite.

	*rMZ* (*SE*)	*nMZ*	*rDZ* (*SE*)	*nDZ*	*n total*
*Ages 7+9+12 composite*	0.63 (0.02)	1099	0.31 (0.02)	1787	2886

Annotation: (rMZ) – correlation between Monozygotic twins, (rDZ) – phenotypic correlation between Dizygotic Twins, (SE) – standard error, (nMZ) – MZ twins sample size, (nDZ) – DZ twins sample size, (n total) – sample size for all individuals.

**Table 2:** Model-fitting estimates for a callous unemotional (CU) trait longitudinal composite.

	*A* (*SE*)	*C* (*SE*)	*E* (*SE*)	*N/pairs*	*N/LL*
*Full ACE model*	0.64 (0.03)	0.00 (0.02)	0.36 (0.02)	2886	3142
*AE model*	0.64 (0.03)	–	0.36 (0.02)	2886	3142

Annotation: (A) – additive genetic, (C) – shared environment, (E) – unique environment, (SE) – standard error, (N/pair) – sample size of twin pairs where both siblings had the phenotypic data, (N/LL) – sample size of twin pairs where at least one sibling had the phenotypic data.

**Table 3:** Fit statistics for structural equation modeling.

	*−2LL*	*df*	*AIC*	*Δχ^2^*	*Δdf*	*p*
*Full ACE model*	15916	5864	4188	–	–	–
*AE model*	15916	5865	4186	−1.86×10^−10^	1	1

Annotation: (−2LL) – minus twice log likelihood of the model, (df) – degrees of freedom, (AIC) – Akaike’s Information Criterion, (Δχ2) – difference between minus twice log likelihoods between the full and the nested model, (Δdf) – difference in degrees of freedom between the full and nested model, (p) – p-value.1.

### GCTA

The GCTA estimate of genetic variance was 0.07, which was not significant given our sample size. The standard error of 0.12 suggests that the proportion of variance explained by the common SNPs tagged by our genome-wide genotypes is highly likely to be less than 20%, which suggests a wide gap with our twin study heritability estimate of 64%. Unlike twin analysis, GCTA does not discriminate C and E because each individual is from a different family. In GCTA, E is a residual term that refers to all variance (including error of measurement) that cannot be attributed to additive genetic effects of the common SNPs included on the DNA array.

### GWA Analysis

**Figure 1:**
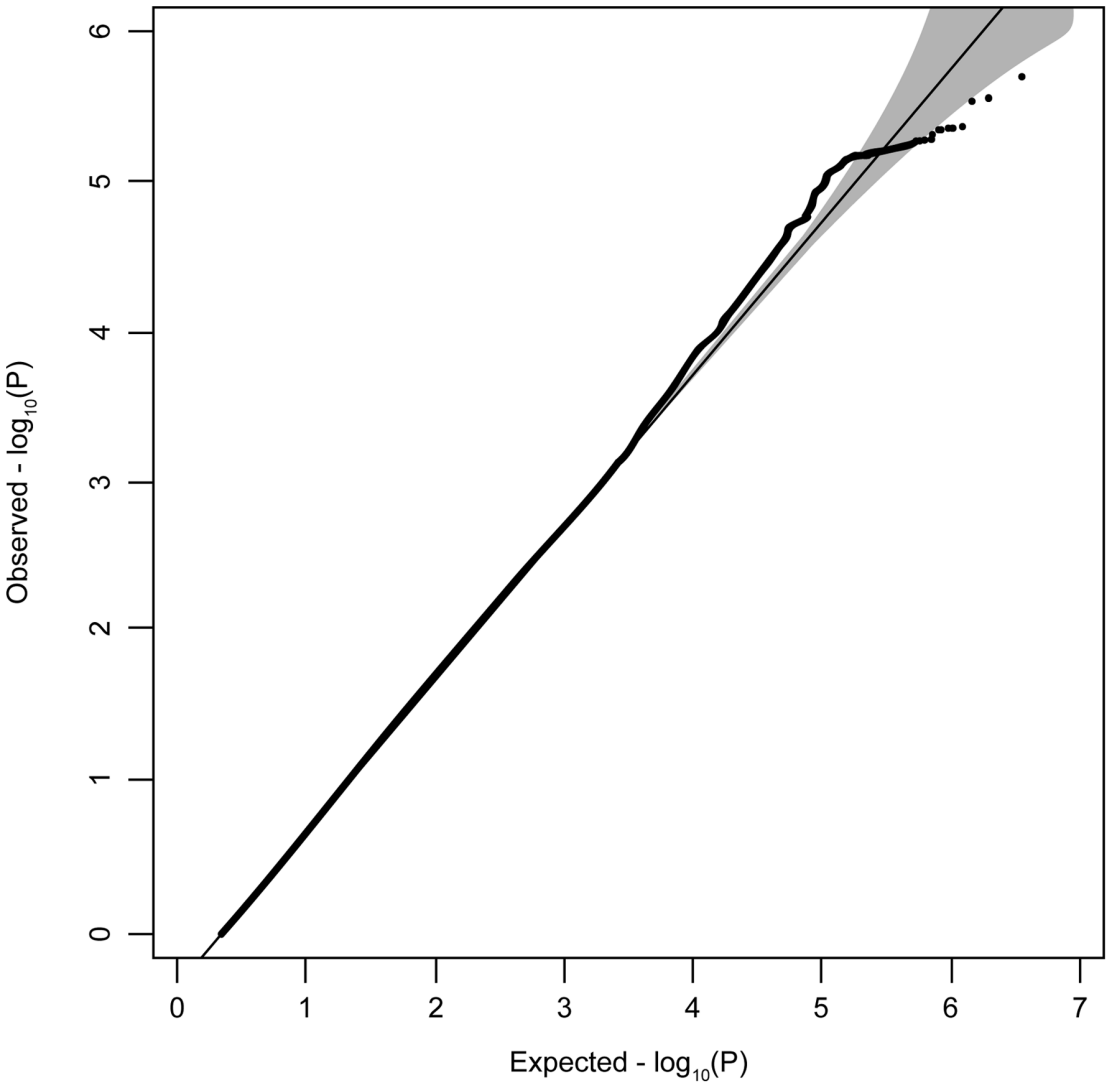
Quantile-quantile plot illustrating the distribution of probability values from the genomewide test of association with CU. X axis: expected quantile of minus log probability values under the null hypothesis. Y axis: observed quantile of minus log probability values for association after adjustment by genomic control. The straight line at x = y represents the null distribution and the gray area surrounding the line indicates a 95% confidence band around the null.

We tested 699,388 genotyped autosomal SNPs and 1,024,929 imputed autosomal SNPs that passed quality control thresholds. The analysis included 2,930 children with admissible data for both genotype and CU. The quantile-quantile (QQ) plot presented in Figure 1 illustrates the distribution of probability values from the genomewide test of association with CU. The line indicates the null hypothesis for the relationship between the observed distribution of probability values and results expected by chance alone. The shaded area indicates the 95% confidence interval around the null values. The resemblance of the distribution of observed test statistics to the null distribution indicates that the results are consistent with chance. We observed very little inflation of the test statistics (λ<1.01).

No single SNP achieved genomewide significance using a conventional significance threshold of *p*<5×10^−8^ [65]. The results for three SNPs achieving suggestive significance at the less stringent threshold of *p*<5×10^−6^ are summarized in Table 4.

**Table 4:** SNPs associated with CU exceeding a threshold for suggestive significance of *p*<5×10^−6^.

*SNP*	*Chromosome*	*Position*	*Imputed*	*Reference/risk allele*	*Risk allele frequency*	*p*	*Genes within 500 Kb*
rs12551906	9	101158911	Yes	A/G	0.689	2.93E-06	SEC61B, ALG2, TGFBR1
rs10865864	3	3603981	No	A/G	0.197	4.11E-06	LRRN1
rs151997	5	50268956	No	C/T	0.382	4.62E-06	PARP8

Alleles are given relative to positive strand as defined by NCBI human genome assembly b36. The “risk” allele is associated with higher CU at 7, 9, and 12; the “reference” allele is the other allele. P =  probability value for multivariate score test after correction by genomic control.

The sample size of 2,930 would, in a univariate analysis, be sufficient to detect a quantitative trait locus (QTL) explaining 1.0% of the variance with 49% power, or a QTL explaining 1.3% of the variance with 77% power [66]. Individual SNP variants with lower frequencies, being yet rarer in the population, are also unlikely to explain such large proportions of variance. The balance of probabilities therefore suggest that there are no autosomal SNPs with large effects on CU (>1% of variance).

## Discussion

Our twin study heritability estimate of 64% for CU is consistent with previously published results [9]. In contrast, this first GCTA estimate of CU heritability based on DNA alone was only 7% – much lower than expected given that it was calculated using an identical measure in the same sample.

Although our sample size of nearly 3000 children entails a large standard error (0.12) for our GCTA heritability estimate (0.07), the 95% confidence interval suggests that the true estimate is GCTA heritability is less than 20%. In other words, even if the true GCTA heritability estimate for CU were at the top of this confidence interval, it would imply a wide gap between the GCTA and twin heritability estimates. This gap between GCTA heritability and twin study heritability could be called ‘missing GCTA heritability’. The missing GCTA heritability gap for CU, if true, would greatly increase the difficulty of identifying GWA associations because GWA, like GCTA, is limited to the additive effects tagged by the common SNPs on our DNA array.

Why is missing GCTA heritability so much greater for CU than for some other traits in the same study? Although it is possible that the twin study overestimated heritability for CU, converging evidence from several sources suggests that twin estimates are valid [39]. Another possibility is that our low GCTA heritability estimate for CU is in error. This seems unlikely for two reasons. First, in the same study using the same methods, we have found GCTA heritability estimates that were more than half the twin study heritability estimate for cognitive and anthropometric traits in the same sample [45]. For example, heritability estimates for height and intelligence were 0.80 and 0.46, respectively, in twin analyses and 0.42 and 0.35 in GCTA. Second, although our GCTA heritability estimate for CU is the first such estimate, our low estimate is similar to the low GCTA heritability estimate reported in the only published GCTA study of personality [44].

What we do know for certain is that GCTA is limited to additive effects of common DNA variants that are tagged by commercially available DNA arrays, whereas the twin design is not. Because identical twins are identical in terms of all inherited DNA sequence variants, they share all genetic effects – small or large, nonadditive or additive, rare or common. For this reason, as compared to twin studies, GCTA underestimates heritability to the extent that heritability is caused by nonadditive genetic effects and the effects of rare variants [67].

The first hypothesis – that nonadditive genetic effects led to the low GCTA estimate of heritability for CU – is not supported by our twin results. As mentioned in the Methods section, the hallmark of nonadditive gene-gene (epistatic) interactions is that the DZ twin correlation is less than half the MZ twin correlation. However, in our twin analysis of CU, the DZ correlation (0.31) is almost exactly half the MZ correlation (0.63), providing no support for the hypothesis of nonadditive genetic influence.

Our twin model-fitting also found that the additive AE model best fit the data. Indirect support for suggesting that nonadditive genetic variance may not be a major factor causing missing GCTA heritability for CU comes from the general conclusion from quantitative genetic research that most genetic variance is additive [39] [68]. There are also evolutionary reasons to expect that most genetic variance is additive [69]. We hope that nonadditive genetic variance is not a major factor because if heritability is substantially due to nonadditive genetic effects, it will be extremely difficult to identify these effects because power is greatly diminished with each gene added to the interaction.

The second hypothesis –less common DNA variants contribute to the GCTA heritability gap for CU – seems certain to be part of the general explanation for missing heritability [70]. Common SNPs on currently available commercial DNA arrays have frequencies greater than one percent in the population. Many more SNPs are rarer, with frequencies that go down to ‘private mutations’ unique to an individual. More than 10 million SNPs have been validated in populations around the world; only about 2 million have frequencies greater than one percent in the population studied. However, we can offer no speculation why rare variants would be so especially important for CU. In order to explain a large proportion of phenotypic variation, rare variants would need to have large effect sizes or be highly numerous. Large effect sizes could occur for a trait under negative selection pressure, in which a de novo mutation has a large effect on the individual who harbors the mutation, but whose fertility is lowered so that the mutation does not spread in the population. Yet schizophrenia, a disorder that is known to be associated with low fecundity and therefore presumably under negative selection pressure, is nevertheless largely influenced by common polygenic variation [41] [71].

Because CU seems unlikely to be under strong negative selection, rare variants of large effect seem an unlikely hypothesis as to why the GCTA heritability gap is so much greater for CU than for other traits.

Whatever its cause, the low GCTA heritability estimate, if true, implies that identifying DNA variants responsible for the heritability of CU will be even more difficult than it is for most complex traits. Because GCTA heritability, not twin study heritability, creates a ceiling for GWA analysis, the low GCTA estimate for CU doomed our GWA from the outset, even beyond the relatively small sample size that was limited to detecting genome-wide significant additive effects tagged by common variants that yield associations accounting for more than 1% of the total variance. This first genome-wide association study of childhood CU in a community sample of 2,930 individuals found no associations that met stringent genomewide correction for multiple testing (*p*<5×10^−8^). Three SNPs achieved suggestive significance at a less stringent threshold (*p*<5×10^−6^), none of which are close to the coding regions of well-documented candidate genes implicated in previous genetic association studies of CU, or any of the suggestive hits identified by the previous pooling study of extreme CU that co-occurred with conduct problems [31].

These results suggest that, for CU in particular, not only will much larger samples be needed to detect associations that account for very small effect sizes, but that methods to identify gene-gene interactions and whole-genome sequencing to detect rare variants may be needed in order to detect DNA variants that are responsible for the heritability of CU.
