# Correction: Genetics of Callous-Unemotional Behavior in Children

**DOI:** 10.1371/annotation/0b16418f-ceb5-41b2-be2a-a20f0c56f9a6

**Published:** 2013-07-16

**Authors:** Essi Viding, Thomas S. Price, Sara R. Jaffee, Maciej Trzaskowski, Oliver S. P. Davis, Emma L. Meaburn, Claire M. A. Haworth, Robert Plomin

The published Financial Disclosure is missing funding information. The following is the correct funding information:

We gratefully acknowledge the ongoing contribution of the parents and children in the Twins Early Development Study (TEDS). TEDS is supported by a program grant [G0901245; and previously G0500079] from the U.K. Medical Research Council (MRC), with additional support from the US National Institutes of Health [HD044454; HD046167 HD059215]. Genome-wide genotyping was made possible by grants from the Wellcome Trust Case Control Consortium 2 project [085475/B/08/Z; 085475/Z/08/Z]. RP is supported by a Medical Research Council Research Professorship award [G19/2] and a European Advanced Investigator award [295366]; O.S.P.D. is supported by a Sir Henry Wellcome Fellowship from the Wellcome Trust [WT088984]; C.M.A.H. is supported by a research fellowship from the British Academy. We also thank the Wellcome Trust Case Consortium 2 team for their help with genotyping and quality control of our data. 

